# A radiomics nomogram for the prediction of overall survival in patients with hepatocellular carcinoma after hepatectomy

**DOI:** 10.1186/s40644-020-00360-9

**Published:** 2020-11-16

**Authors:** Qinqin Liu, Jing Li, Fei Liu, Weilin Yang, Jingjing Ding, Weixia Chen, Yonggang Wei, Bo Li, Lu Zheng

**Affiliations:** 1 Department of Liver Surgery, Center of Liver Transplantation, West China Hospital, Sichuan University, 37 Guo Xue Road, Chengdu, 610041 Sichuan Province China; 2 Department of Hepatobiliary Surgery, the Second Affiliated Hospital of Army Medical University, No. 183 Xinqiao High Street, Shapingba District, Chongqing, 400037 China; 3 The First Affiliated Hospital of Jinan University, Guangzhou, China; 4 Department of Radiology, West China Hospital, Sichuan University, Chengdu, China; 5 Department of Gastrointestinal Surgery, West China Hospital, Sichuan University, Chengdu, China

**Keywords:** CT texture analysis, Hepatocellular carcinoma, Overall survival, Prediction, Nomogram

## Abstract

**Background:**

Hepatocellular carcinoma (HCC) is associated with a dismal prognosis, and prediction of the prognosis of HCC can assist in therapeutic decision-makings. An increasing number of studies have shown that the texture parameters of images can reflect the heterogeneity of tumors, and may have the potential to predict the prognosis of patients with HCC after surgical resection. The aim of this study was to investigate the prognostic value of computed tomography (CT) texture parameters in patients with HCC after hepatectomy and to develop a radiomics nomogram by combining clinicopathological factors and the radiomics signature.

**Methods:**

In all, 544 eligible patients were enrolled in this retrospective study and were randomly divided into the training cohort (*n* = 381) and the validation cohort (*n* = 163). The tumor regions of interest (ROIs) were delineated, and the corresponding texture parameters were extracted. The texture parameters were selected by using the least absolute shrinkage and selection operator (LASSO) Cox model in the training cohort, and a radiomics signature was established. Then, the radiomics signature was further validated as an independent risk factor for overall survival (OS). The radiomics nomogram was established based on the Cox regression model. The concordance index (C-index), calibration plot and decision curve analysis (DCA) were used to evaluate the performance of the radiomics nomogram.

**Results:**

The radiomics signature was formulated based on 7 OS-related texture parameters, which were selected in the training cohort. In addition, the radiomics nomogram was developed based on the following five variables: α-fetoprotein (AFP), platelet-to-lymphocyte ratio (PLR), largest tumor size, microvascular invasion (MVI) and radiomics score (Rad-score). The nomogram displayed good accuracy in predicting OS (C-index = 0.747) in the training cohort and was confirmed in the validation cohort (C-index = 0.777). The calibration plots also showed excellent agreement between the actual and predicted survival probabilities. The DCA indicated that the radiomics nomogram showed better clinical utility than the clinicopathologic nomogram.

**Conclusion:**

The radiomics signature is a potential prognostic biomarker of HCC after hepatectomy. The radiomics nomogram that integrated the radiomics signature can provide a more accurate estimation of OS than the clinicopathologic nomogram for HCC patients after hepatectomy.

**Supplementary Information:**

The online version contains supplementary material available at 10.1186/s40644-020-00360-9.

## Background

HCC is the fifth most common malignancy and ranks as the third most common cause of cancer-related death worldwide [1, 2]. Surgical resection is the preferred treatment option for individuals with HCC [3]. However, the long-term prognosis of patients with hepatocellular carcinoma after resection is dismal, as the 5-year survival rate is only 25–55% and the 5-year recurrence rate is 60-100% [4–7]. The prognosis of HCC is influenced by numerous factors, and thus, early prediction of the prognosis is of great significance for the long-term management and effective treatment of patients with this disease. Currently, the Barcelona Clinic Liver Cancer (BCLC) system is the most recognized staging system for HCC worldwide and is a widely used tool that guides prognostic prediction and treatment decisions [3]. Despite this, the BCLC classification is still controversial and has limited predictive power [8–10]. Therefore, it is worthwhile to explore additional reliable and pragmatic methods that can be used to evaluate the prognosis of HCC.

Previous imaging studies were based on the shape, density and enhancement of tumors [11], which did not quantify the information of the images and were easily affected by the subjectivity of the radiologists. It is known that malignant tumors are composed of heterogeneous cells and their surrounding microenvironment, and that intra-tumor heterogeneity is associated with tumor angiogenesis and biological behavior, which can be assessed through imaging traits. Radiomics is an emerging field, in which high-dimensional information can be extracted from medical images. Texture analysis (TA), an image post-processing technique, can be used to evaluate the potential heterogeneity of lesions based on a large set of quantitative features [12–14]. Emerging studies have shown that texture features have the potential to differentiate tumor types, monitor therapeutic response, identify regional lymph node metastasis of malignant tumors and predict prognosis [15, 16]. Huang et al. [17] developed a radiomics nomogram, which exhibited favorable accuracy for the preoperative prediction of lymph node metastasis in patients with colorectal cancer. Wu et al. [18] developed a radiomics nomogram for the preoperative prediction of lymph node metastasis in bladder cancer and found that CT texture parameters were independent predictors of response to chemotherapy. Ahn et al. [19] demonstrated that lower skewness in the 2D analysis and a narrower SD in the 3D analysis were useful predictors of chemotherapeutic response in patients with colon cancer liver metastasis. In addition, the radiomics signature could be used to predict preoperative individualized MVI status and early recurrence of HCC [20, 21]. Furthermore, previous studies have shown that radiomics features are correlated with gene expression, gene mutations and epigenetic alterations through capturing the tumor phenotypes, which are associated with underlying gene expression patterns of cancer and may reflect cellular proliferation, invasion, metastasis and drug resistance of the tumors [22–26]. Segal et al. [23] reported that variations in 116 gene modules can be reconstructed from 28 imaging traits. However, due to the limited number of studies, additional studies are needed to confirm the potential association between radiophenotype and gene expression [27, 28]. In the future, accurate and quantitative imaging information based on an artificial intelligence automatic image recognition and diagnosis system in combination with clinical data can help doctors evaluate patient survival. It plays an important role in clinical decision making, treatment planning and postoperative long-term follow-up, and provides new opportunities for individual precise treatment.

The underlying correlation among radiomics features, pathology and survival is not clear, and relatively few studies have addressed the efficacy of TA in prognostic prediction. The intratumor heterogeneity can reflect the biological characteristics of the tumor, which may be of prognostic significance. The purpose of this study is to explore the prognostic value of preoperative CT texture parameters for patients who underwent radical hepatectomy. In addition, a prognostic nomogram is proposed on the basis of texture parameters to provide useful references for precision medicine.

## Methods

### Patients

In all, 544 consecutive patients with HCC who underwent hepatectomy in the Department of Liver Surgery at West China Hospital between January 2013 and December 2016 were enrolled according to the following inclusion criteria: (1) patients who underwent initial radical hepatectomy with pathologically confirmed HCC; (2) Child-Pugh A or B liver function; (3) no preoperative treatments such as radiofrequency ablation, transcatheter arterial chemoembolization (TACE) and chemotherapy; and (4) preoperative contrast-enhanced CT performed within 4 weeks. The exclusion criteria were as follows: (1) CT images with invisible lesions or severe artifacts; (2) patients with benign or mixed types of liver tumor; (3) those who underwent simultaneous hepatectomy and radiofrequency ablation; (4) liver transplantation performed during the course of disease; (5) incomplete clinical or follow-up data. We randomly divided the eligible patients at a ratio of 7:3 into 2 groups: the training cohort (*n* = 381) and the validation cohort (*n* = 163). This study was approved by the Committee of Ethics of West China Hospital of Sichuan University. The clinicopathologic variables were collected, including patient demographics, laboratory data, tumor characteristics, surgical outcomes, and postoperative pathological data.

### Patient follow-up and surveillance

All patients were followed-up by telephone or outpatient visit during the first month after surgery and then every 3 months thereafter until November 2019. The routine examinations, which included serum AFP levels, routine blood tests, serum biochemistry, hepatitis B virus deoxyribonucleic acid (HBV-DNA), abdominal ultrasonography and contrast-enhanced CT/MRI, were performed at each outpatient follow-up visit. OS was calculated as the period from the time of surgery to the time of either death or last follow-up.

### Image acquisition and imaging texture analysis

All CT scans of the liver were acquired on a Siemens scanner (Siemens Somatom Definition FLASH, Siemens Healthcare, Erlangen, Germany). After 6 h of fasting, patients received an intravenous administration of 1.5 ml/kg of Iohexol (Jiangsu Yangtze River Pharmaceutical Group Co., Ltd., Taizhou, China; 300 mg of iodine/ml) at a rate of 2–3 ml/s. Then, images acquisition was performed in the arterial phase and venous phase at 25 and 70 s, respectively. The following scan parameters were used: tube voltage of 120–140 kV, tube current of 210 mA, pitch of 4.0, matrix size of 512 × 512, slice thickness of 5 mm, and a high spatial resolution algorithm.

The target images were retrieved from the Picture Archiving and Communication System in Digital Imaging and Communications in Medicine (DICOM) format and transferred to Mazda software (version 4.6) for further TA. All manual segmentations were performed by an abdominal radiologist with 5 years of experience and were verified by a senior radiologist with 20 years of experience. The two-dimensional regions of interest (ROIs) delineated the largest cross-sectional area of the tumors on the preoperative portal venous phase images. For patients with multifocal HCCs, the ROIs of the largest tumor were selected for further analysis. The CT images on the portal venous phase were used for radiomics feature extraction because hypovascular HCC may influence the accuracy and reproducibility of the ROIs delineation on the arterial phase [29], and the previous studies showed the excellent predictive performance of texture features in this phase [30, 31].

According to the segmented tumors, 270 texture features that reflected tumor heterogeneity were extracted, including the following 5 categories: (1) histogram features; (2) co-occurrence matrix; (3) run-length matrix; (4) autoregressive model; (5) wavelet transform [32]. Detailed information on the texture features is available in Supplementary Table S1.

### Feature selection and radiomics signature building

We used the LASSO Cox regression model to select the features that were most associated with the survival status of the training cohort, and a 10-fold cross validation was used to reduce overfitting [33]. LASSO is a data analysis method that can shrink the coefficients of variables unrelated to survival to zero, and thus, the features with non-zero coefficient were selected [34]. The optimal tuning parameter was determined by minimum criteria (minimum lambda). The radiomics signature was built via a linear combination of selected features multiplied by their corresponding non-zero coefficients. Then, the Rad-score was calculated for each patient.

### Model construction and evaluation

The patients were stratified into either the high-risk or low-risk groups according to the threshold of the Rad-score calculated by ROC curve analysis. The difference between the survival curves of the high-risk and low-risk groups was assessed in the training cohort and then validated in the validation cohort. Univariate and multivariate Cox regression analyses were performed in the training cohort to determine the potential independent risk factors. Then a radiomics nomogram that integrates the radiomics signature and the independent clinicopathological risk factors according to the result of the multivariate analysis was constructed to predict postoperative survival status. The discrimination ability of the nomogram was evaluated using the C-index. The calibration performance was measured by the calibration curve, which described the agreement between the predicted and observed survival probability. The clinical value of the nomogram was assessed in the whole cohort by DCA [35], which was generated by calculating the net benefits at different threshold probabilities.

### Statistical analysis

The statistical analysis was performed with SPSS version 22.0 software (Chicago, IL, USA) and R software (version 3.5.1; **http://www.R-project.org**). Continuous variables were presented as the mean ± standard deviation for normally distributed variables or as the median (interquartile range) for non-normally distributed variables. Differences between the two groups were compared using the *t*-test or Mann-Whitney *U* test. Additionaly, categorical variables were expressed by frequency (percentage) and assessed by Pearson’s chi-square test or Fisher’s exact test. ROC curve analysis was used to determine the optimal cutoff values based on the maximum Youden index. Survival curves were calculated using the Kaplan-Meier method and were compared using the log-rank test. The Cox regression analysis was used for both univariate and multivariate analyses. Variables with *P*-values < 0.10 in the univariable analysis were introduced into the multivariate Cox proportional hazards model to further determine the independent prognostic factors with a backward stepwise selection. The LASSO Cox regression model analysis was based on the glmnet package. The nomogram and calibration curve were established using the rms package, while the DCA was performed using the dca. R package. The predictive performance of the nomograms was evaluated by the C-index and was compared using the Rcorrp.cens package in Hmisc in R. A *P* value < 0.05 was considered statistically significant.

## Results

### Patient demographics and clinicopathological characteristics

In all, 544 patients who met the inclusion criteria were retrospectively analyzed. The comparison of the clinicopathological characteristics between the training cohort (*n* = 381) and the validation cohort (*n* = 163) is shown in Table 1. The median follow-up time was 28.8 months (range, 15.1–40.5 months) in the training cohort and 27.2 months (range, 16.9–39.5 months) in the validation cohort. No significant differences were observed in the baseline characteristics between the two groups (*P* > 0.05), which suggests similarity between the cohorts.

**Table 1:** Clinicopathological factors of 544 patients who underwent radical hepatectomy

Variables	Training cohort (*n* = 381)	Validation cohort (*n* = 163)	*P*
Age, years	51.3 ± 11.2	50.2 ± 11.5	0.301
Sex			0.410
Male	324 (85.0%)	143 (87.7%)	
Female	57 (15.0%)	20 (12.3%)	
BMI, Kg/m^2^			0.713
< 18.5	27 (7.1%)	12 (7.4%)	
18.5–25	258 (67.7%)	116 (71.2%)	
≥ 25	96 (25.2%)	35 (21.5%)	
HBsAg			0.327
Positive	328 (86.1%)	135 (82.8%)	
Negative	53 (13.9%)	28 (17.2%)	
HBV-DNA (copies/ml)			
< 10^3^	176 (46.2%)	79 (48.5%)	0.645
≥ 10^3^	205 (53.8%)	84 (51.5%)	
Liver cirrhosis			0.142
Present	263 (69.0%)	102 (62.6%)	
Absent	118 (31.0%)	61 (37.4%)	
Child-Pugh classification			0.204
A	367 (96.3%)	161 (98.8%)	
B	14 (3.7%)	2 (1.2%)	
Previous abdominal surgery			0.705
Present	61 (16.0%)	24 (14.7%)	
Absent	320 (84.0%)	139 (85.3%)	
Comorbidities			0.012
Present	73 (19.2%)	17 (10.4%)	
Absent	308 (80.8%)	146 (89.6%)	
AFP, ng/mL			0.091
< 400	233 (61.2%)	87 (53.4%)	
≥ 400	148 (38.8%)	76 (46.6%)	
CEA, ng/mL			0.575
Normal	297 (78.0%)	131 (80.4%)	
Abnormal	84 (22.0%)	32 (19.6%)	
CA19–9, U/ml			0.588
Normal	233 (61.2%)	104 (63.8%)	
Abnormal	148 (38.8%)	59 (36.2%)	
TBIL, umol/L	14.0 (10.9–17.8)	13.7 (11.0–18.4)	0.794
DBIL, umol/L	5.4 (4.1–6.8)	5.3 (4.2–6.8)	0.853
ALT, IU/L	38.0 (27.0–56.8)	39.0 (25.0–62.0)	0.974
AST, IU/L	38.0 (30.0–58.0)	39.0 (30.0–59.0)	0.875
Albumin, g/L			0.909
<35	27 (7.1%)	12 (7.4%)	
≥ 35	354 (92.9%)	151 (92.6%)	
NLR	2.2 (1.7–3.1)	2.3 (1.6–3.2)	0.776
PLR	92.9 (65.1–128.3)	84.7 (64.0–135.9)	0.
ASA grade			0.088
II	316 (82.9%)	125 (76.7%)	
III	65 (17.1%)	38 (23.3%)	
Largest tumor size, cm	5.0 (3.2–7.8)	5.6 (3.4–9.0)	0.106
Tumor number			0.965
Solitary	358 (94.0%)	153 (93.9%)	
Multiple	23 (6.0%)	10 (6.1%)	
Hepatectomy			0.297
Anatomical	213 (55.9%)	99 (60.7%)	
Nonanatomical	168 (44.1%)	64 (39.3%)	
Hemorrhage, ml			0.498
<200	133 (34.9%)	52 (31.9%)	
≥ 200	248 (65.1%)	111 (68.1%)	
Intraoperative transfusion			0.606
Yes	30 (7.9%)	15 (9.2%)	
No	351 (92.1%)	148 (90.8%)	
Differentiation			0.927
poor	169 (44.4%)	73 (44.8%)	
Well-moderate	212 (55.6%)	90 (55.2%)	
MVI			0.477
Present	124 (32.5%)	48 (29.4%)	
Absent	257 (67.5%)	115 (70.6%)	
Capsule			0.789
Incomplete	215 (56.4%)	94 (57.7%)	
Complete	166 (43.6%)	69 (42.3%)	

*ASA* American Society of Anesthesiologists, *BMI* Body mass index, *AFP* α-fetoprotein, *ALT* Alanine transaminase, *AST* Aspartate aminotransferase, *NLR* Neutrophil-to-lymphocyte ratio, *PLR* Platelet lymphocyte ratio, *MVI* Microvascular invasion

### Construction and validation of the radiomics signature

**Fig. 1:**
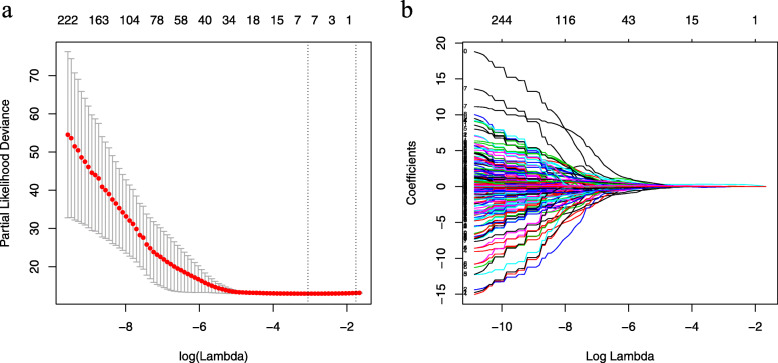
Radiomics feature selection using the LASSO Cox regression model. **a** The partial likelihood deviance was plotted versus log (lambda). The y-axis indicates the partial likelihood deviance, while the lower x-axis indicates the log (lambda) and the upper x-axis represents the average number of predictors. Dotted vertical lines were drawn at the optimal values using the minimum criteria and 1 standard error of the minimum criteria. The tuning parameter (*λ*) was selected in the LASSO model via 10-fold cross-validation based on minimum criteria. **b** LASSO coefficient profiles of the 270 radiomics features. The coefficients (y-axis) were plotted against log (lambda) and 7 features with nonzero coefficients were selected to build the radiomics signature

**Fig. 2:**
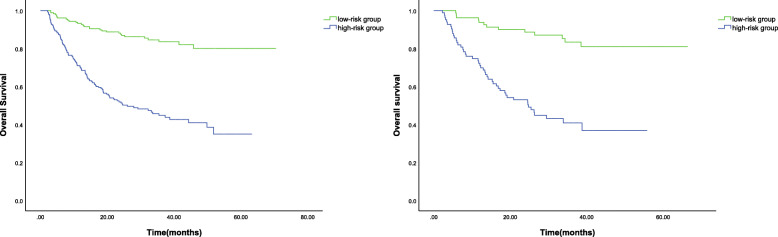
Kaplan-Meier analyses of overall survival according to the risk groups. **a** The overall survival of patients in the high- and low-risk groups in the training cohort. **b** The overall survival of patients in the high- and low-risk groups in the validation cohort

We evaluated the ROIs of hepatic tumors from preoperative CT images and extracted a total of 270 texture features. Then, the LASSO Cox regression model was used to select the most significant features for survival prediction (Fig. 1). When the minimum lambda was 0.034, seven potential predictors of OS-related features with non-zero coefficients were screened out in the training cohort. A radiomics signature was constructed with the selected features and their respective weights. The Rad-score for each patient can be calculated using the following formula: Rad-score = S (0,1) Correlat *0.026 + S (0,3) Correlat*0.036 + Horzl_GLevNonU*0.312 + 45dgr_RLNonUni*0.024 + 45dgr_GLevNonU*0.036 + Sigma*(− 0.068) + WavEnLH_s-4*0.037). According to the optimum cut-off Rad-score based on the maximum Youden index in the training cohort, all patients were classified into the high-risk group (Rad-score ≥ − 0.559) or the low-risk group (Rad-score < − 0.559). The OS was compared between the two groups using a Kaplan-Meier analysis (Fig. 2) in both the training and validation cohorts. The 1, 3 and 5-year OS rates of the low-risk group were 91.7, 82.1 and 78.7%, respectively, which were significantly higher than those of the high-risk group in the training cohort (71.0, 45.5 and 35.5%, *P* < 0.001). The performance of the radiomics signature was confirmed in the validation cohort, and a significant difference was observed in the 1, 3 and 5-year OS rates between the high-risk and low-risk groups (72.3, 40.9, 36.8% vs. 93.8, 83.4, 81.0%, *P* < 0.001). We also observed that patients with lower Rad-scores generally had a better OS.

To further evaluate the association between the radiomics signature and the clinicopathological features, the clinicopathological data of the high-risk and low-risk groups were compared (Table 2). In the training cohort, no significant difference was found between the low-risk and high-risk groups with regard to age, sex, BMI, HBsAg, HBV-DNA, liver cirrhosis, Child-Pugh classification, previous abdominal surgery, comorbidities, CEA, CA19–9, TBIL, DBIL, ALT, Albumin, NLR, ASA grade, tumor number, hepatectomy, and differentiation. However, the high-risk group was positively associated with higher AFP (*P =* 0.007), higher AST (*P* < 0.001), higher PLR (*P* = 0.001), larger tumor size (*P* < 0.001), more hemorrhage (*P* < 0.001), higher incidence of intraoperative transfusion (*P* = 0.019), the presence of MVI (*P* = 0.003), an incomplete tumor capsule (*P* < 0.001) and higher Rad-score (*P* < 0.001).

**Table 2:** The clinicopathological data of patients according to the risk-stratified groups in the training cohort

Variables	high-risk group (*n* = 201)	low-risk group (*n* = 180)	*P*
Age, years	51.7 ± 11.3	50.8 ± 11.0	0.409
Sex			0.551
Male	173 (86.1%)	151 (83.9%)	
Female	28 (13.9%)	29 (16.1%)	
BMI, Kg/m^2^			0.258
<18.5	17 (8.5%)	10 (5.5%)	
18.5–25	129 (64.2%)	130 (72.2%)	
≥ 25	55 (27.4%)	40 (22.2%)	
HBsAg			0.367
Positive	170 (84.6%)	158 (87.8%)	
Negative	31 (15.4%)	22 (12.2%)	
HBV-DNA (copies/ml)			0.068
<10^3^	84 (41.8%)	92 (51.1%)	
≥ 10^3^	117 (58.2%)	88 (48.9%)	
Liver cirrhosis			0.086
Present	131 (65.2%)	132 (73.3%)	
Absent	70 (34.8%)	48 (26.7%)	
Child-Pugh classification			0.738
A	193 (96.0%)	174 (96.7%)	
B	8 (4.0%)	6 (3.3%)	
Previous abdominal surgery			0.285
Present	36 (17.9%)	25 (13.9%)	
Absent	165 (82.1%)	155 (86.1%)	
Comorbidities			0.242
Present	43 (21.4%)	30 (16.7%)	
Absent	158 (78.6%)	150 (83.3%)	
AFP, ng/mL			0.007
< 400	110 (54.7%)	123 (68.3%)	
≥ 400	91 (45.3%)	57 (31.7%)	
CEA, ng/mL			0.543
Normal	159 (79.1%)	138 (76.7%)	
Abnormal	42 (20.9%)	42 (23.3%)	
CA19–9, U/ml			0.639
Normal	121 (60.2%)	112 (62.2%)	
Anormal	80 (39.8%)	68 (37.8%)	
TBIL, umol/L	14 (11.1–18.1)	14.0 (10.7–17.3)	0.948
DBIL, umol/L	5.6 (4.2–7.0)	5.4 (4.0–6.8)	0.375
ALT, IU/L	38.0 (26.0–56.5)	37.0 (27.0–56.5)	0.880
AST, IU/L	43.0 (31.0–72.0)	36.0 (28.0–45.8)	< 0.001
Albumin, g/L			0.483
< 35	16 (8.0%)	11 (6.1%)	
≥ 35	185 (92.0%)	169 (93.9%)	
NLR	2.2 (1.7–3.1)	2.2 (1.7–2.9)	0.113
PLR	92.9 (65.1–128.3)	83.7 (62.6–114.3)	0.001
ASA grade			0.199
II	162 (80.6%)	154 (85.6%)	
III	39 (19.4%)	26 (14.4%)	
Largest tumor size, cm	7.0 (5.0–10.0)	3.7 (2.5–5.0)	<0.001
Tumor number			0.709
Solitary	188 (93.5%)	170 (94.4%)	
Multiple	13 (6.5%)	10 (5.6%)	
Hepatectomy			0.245
Anatomical	118 (58.7%)	95 (52.8%)	
Nonanatomical	83 (41.3%)	85 (47.2%)	
Hemorrhage, ml			< 0.001
< 200	53 (26.4%)	80 (44.4%)	
≥ 200	148 (73.6%)	100 (55.6%)	
Intraoperative transfusion			0.019
Yes	22 (10.9%)	8 (4.4%)	
No	179 (89.1%)	172 (95.6%)	
Differentiation			0.068
poor	98 (48.8%)	71 (39.4%)	
Well-moderate	103 (51.2%)	109 (60.6%)	
MVI			< 0.001
Present	87 (43.3%)	37 (20.6%)	
Absent	114 (56.7%)	143 (79.4%)	
Capsule			< 0.001
Incomplete	137 (68.2%)	78 (43.3%)	
Complete	64 (31.8%)	102 (56.7%)	
Rad-score	−0.1(−0.3 ~ 0.5)	−0.8(−1.0 ~ −0.7)	< 0.001

*ASA* American Society of Anesthesiologists, *BMI* Body mass index, *AFP* α-fetoprotein, *ALT* Alanine transaminase, *AST* Aspartate aminotransferase, *NLR* Neutrophil-to-lymphocyte ratio, *PLR* Platelet lymphocyte ratio, *MVI* Microvascular invasion, *Rad-score* Radiomics score

### Development and validation of the radiomics nomogram

**Fig. 3:**
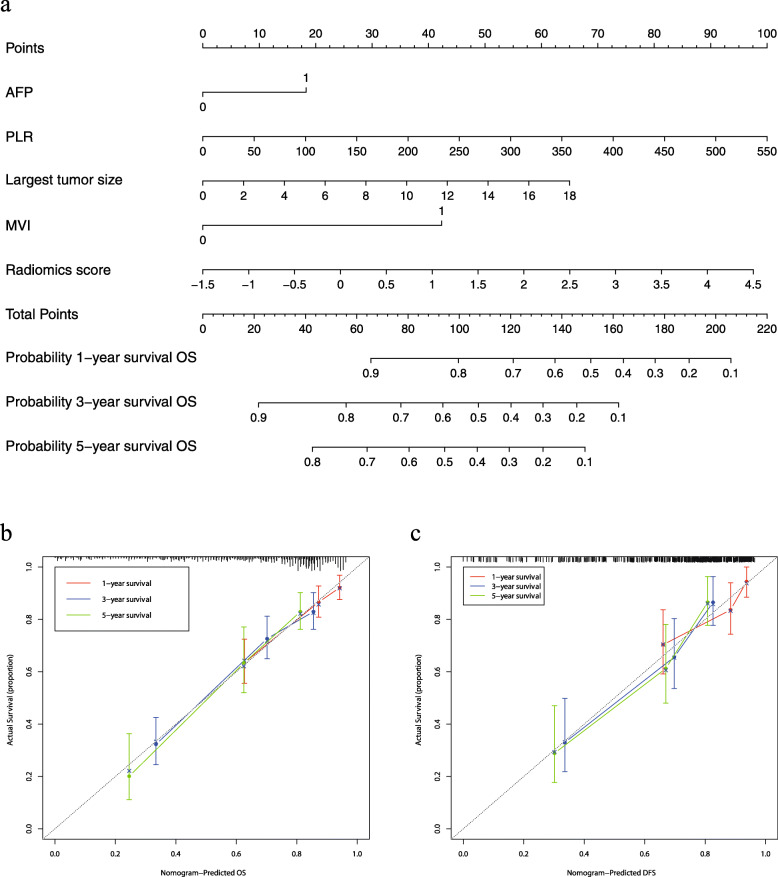
The radiomics nomogram for the prediction of survival status (**a**). The calibration curves of the radiomics nomogram in the training cohort (**b**) and the validation cohort (**c**)

The results of the univariate analysis based on the training cohort are displayed in Table 3. According to the univariate analysis, HBsAg, HBV-DNA, AFP, NLR, PLR, largest tumor size, hemorrhage, intraoperative transfusion, differentiation, MVI, capsule and Rad-score were potential risk factors for OS. However, the results of the multivariate analysis suggested that only AFP (HR 1.566; CI 1.101–2.226; *p* = 0.013), PLR (HR 1.004; CI 1.001–1.007; *p* = 0.010), largest tumor size (HR 1.084; CI 1.027–1.145; *p* = 0.003), MVI (HR 2.509; CI 1.751–3.594; *p*<0.001) and Rad-score (HR 1.398; CI 1.188–1.646; *p*<0.001) were independently associated with an unfavorable postoperative survival. The radiomics nomogram was constructed with the 5 independent risk predictors identified above to predict the personalized survival status of the patients, while the clinicopathologic nomogram incorporated only the independent clinicopathological risk factors. The C-index of the clinicopathologic nomogram was 0.726 (95% CI 0.705–0.748) in the training cohort and 0.720 (95% CI 0.686–0.755) in the validation cohort. The radiomics nomogram yielded a C-index of 0.747 (95% CI, 0.727–0.768) in the training cohort and 0.777 (95% CI, 0.748–0.806) in the validation cohort. The radiomics nomogram showed improved discrimination performance when the radiomics signature was integrated into the clinicopathologic nomogram (*P* = 0.002 in the training cohort, *p* < 0.001 in the validation cohort; Table 4). The radiomics nomogram and the corresponding calibration curve are presented in Fig. 3. The calibration curve demonstrated satisfactory consistency between the nomogram-predicted survival and the actual observed survival in both the training and validation cohorts.

**Table 3:** Univariate and multivariate Cox regression analyses for patients in the training cohort

Variables	Univariate analysis		Multivariate analysis	
	HR (95%CI)	*p*	HR (95%CI)	*p*
Age, years	0.986 (0.971–1.000)	0.052		
Sex, male vs. female	1.675 (0.964–2.911)	0.067		
BMI, Kg/m^2^				
<18.5vs.18.5–25	0.944 (0.455–1.957)	0.876		
25vs.18.5–25	1.171 (0.539–2.541)	0.691		
HBsAg, Positive vs. Negative	1.950 (1.079–3.523)	0.027		
HBV-DNA, copies/ml, <10^3^ vs. ≥ 10^3^	1.740 (1.223–2.476)	0.002		
Liver cirrhosis, Present vs. Absent	0.826 (0.583–1.171)	0.283		
Child-Pugh classification, B vs. A	1.684 (0.825–3.438)	0.152		
Previous abdominal surgery, Present vs. Absent	0.893 (0.562–1.420)	0.632		
Comorbidities, Present vs. Absent	1.016 (0.668–1.546)	0.941		
AFP, ng/mL, ≥ 400vs.<400	1.931 (1.388–2.688)	< 0.001	1.566 (1.101–2.226)	0.013
CEA, ng/mL, Normal vs. Abnormal	0.656 (0.419–1.027)	0.065		
CA19–9, U/ml, Normal vs. Abnormal	1.131 (0.807–1.585)	0.474		
TBIL, umol/L	1.000 (0.988–1.013)	0.940		
DBIL, umol/L	0.999 (0.982–1.016)	0.901		
ALT, IU/L	1.000 (0.999–1.002)	0.823		
AST, IU/L	1.000 (0.999–1.002)	0.423		
Albumin, g/L, <35vs. ≥ 35	1.322 (0.732–2.390)	0.355		
NLR	1.085 (1.030–1.143)	0.002		
PLR	1.006 (1.004–1.009)	< 0.001	1.004 (1.001–1.007)	0.010
ASA grade, III vs. II	1.362 (0.910–2.039)	0.133		
Largest tumor size, cm	1.193 (1.145–1.244)	< 0.001	1.084 (1.027–1.145)	0.003
Tumor number, Solitary vs. Multiple	1.314 (0.710–2.432)	0.384		
Hepatectomy, Anatomical vs. Nonanatomical	0.755 (0.538–1.060)	0.105		
Hemorrhage, ml, ≥ 200vs.<200	1.927 (1.310–2.836)	0.001		
Intraoperative transfusion, Yes vs. No	1.860 (1.104–3.132)	0.020		
Differentiation, poor vs. Well-moderate	1.598 (1.148–2.225)	0.006		
MVI, Present vs. Absent	3.524 (2.524–4.921)	<0.001	2.509 (1.751–3.594)	<0.001
Capsule, Incomplete vs. Complete	1.891 (1.324–2.702)	< 0.001		
Rad-score	1.493 (1.324–1.684)	< 0.001	1.398 (1.188–1.646)	<0.001

*ASA* American Society of Anesthesiologists, *BMI* Body mass index, *AFP* α-fetoprotein, *ALT* Alanine transaminase, *AST* Aspartate aminotransferase, *NLR* Neutrophil-to-lymphocyte ratio, *PLR* Platelet lymphocyte ratio, *MVI* Microvascular invasion, *Rad-score* Radiomics score

**Table 4:** Performance of the radiomics and clinicopathologic nomogram for Prediction of OS

Nomogram	The training cohort		The validation cohort	
	C-index	*P*-value	C-index	*P* -value
Radiomics	0.747 (0.727–0.768)	0.002	0.777 (0.748–0.806)	< 0.001
Clinicopathological	0.726 (0.705–0.748)		0.720 (0.686–0.755)

### Clinical utility

**Fig. 4:**
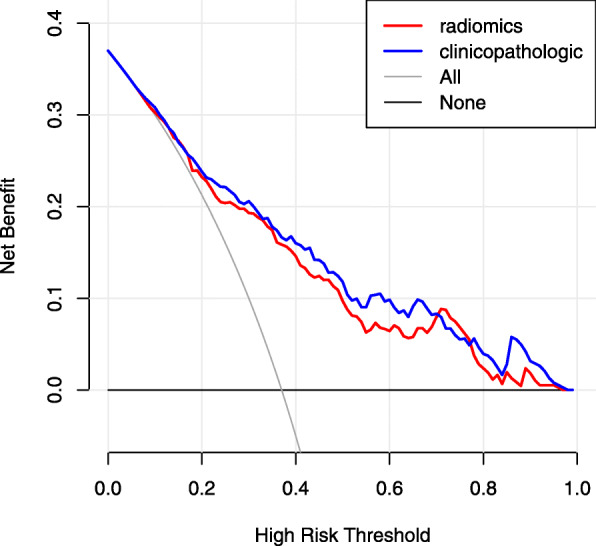
Decision curve analysis of the radiomics and clinicopathologic nomogram in the entire cohort (*n* = 544). The y-axis represents the net benefit, and the x-axis represents the threshold probability. The black line represents the assumption that no patients exhibited long-term overall survival (OS). The grey line represents the assumption that all patients exhibited long-term OS. The decision curves indicated that the radiomics nomogram (red line) showed better clinical utility than the clinicopathologic nomogram (blue line)

The DCA of the radiomics and clinicopathologic nomograms is presented in Fig. 4. The net benefit was calculated by adding the true positives and subtracting the false positives. The straight line represents the assumption that all patients will die, and the horizontal line represents the assumption that no patients will die. The DCA demonstrated that the nomograms added more net benefit compared with the treat-all strategy or treat-none strategy with a threshold probability of 10% or greater. Moreover, the radiomics nomogram provided a higher net benefit than the clinicopathologic nomogram in terms of survival prediction in HCC patients.

## Discussion

Surgical resection is the mainstay curative treatment for individuals with HCC, but the prognosis varies from patient to patient. The prediction of survival status in patients with HCC after surgery is important for clinical decision-making. Among the numerous prognostic factors, tumor heterogeneity is one of the most important contributions, which may relate to different natural histories, environmental susceptibility and individual genetic tendencies [36]. Intra-tumoral heterogeneity can reveal tumor growth, metastatic potential and response to treatment and may thus be a potential prognostic predictor of disease outcome [37]. However, previous studies were mainly based on clinicopathological factors [38, 39] and either seldom involved imaging information or only considered a small number of subjective imaging parameters [40, 41]. Moreover, a large amount of tumor-related information that can be extracted from images is ignored. Radiomics can capture the potential heterogeneity of lesions using a large number of quantitative imaging features, which may be a valuable supplement to the existing predictors.

Medical imaging plays an important role in preoperative diagnosis, choice of therapy, therapeutic effect evaluation and disease surveillance. However, the interpretation of medical imaging is often based on physicians’ personal expertise and experience, which are subjective and qualitative. Radiomics can be used to analyze the texture parameters extracted by a computer and can allow the quantitative assessment of the pixel differences in images to provide more comprehensive information about tumors that may not be detected by the human eye. In addition, the temporal and spatial heterogeneity of the tumor can be evaluated by whole tumor analysis instead of in limited biopsy samples [42]. Medical imaging analysis can reveal the tumor biological processes and microenvironment characteristics and may assist in therapeutic decision-making. However, few studies have focused on the prognostic prediction in patients with HCC. Therefore, this study aimed to develop a radiomics signature to predict the prognosis of patients with HCC after surgical resection based on selected radiomics features. Moreover, a nomogram was constructed based on the independent risk factors, which allows for more precise prognostication, better clinical management and more appropriate adjuvant therapy. This study introduced a noninvasive, low cost and reproducible method to predict the outcomes in patients with resectable HCC, which is of great significance for personalized medicine.

In our study, 5 optimal features were selected from 270 radiomics features of the portal venous phase via the LASSO method to build a radiomics signature, after which the patients were divided into the high-risk and low-risk groups according to the Rad-score threshold. The results indicate that patients with higher Rad-scores were more likely to have a worse OS than those with lower Rad-scores. In the multivariate analysis, the radiomics signature was further demonstrated to be an independent predictor of OS. This study provides a method for prognosis-related high-dimensional data selection. LASSO is a penalized regression approach that selects covariates with non-zero coefficients among numerous covariates to avoid overfitting, thus improving the prediction efficiency [43]. Those radiomic features provided a quantitative description of the position, intensity and inter-relationship of the pixels [12, 44] to reveal tumor phenotypic differences and to evaluate the intra-tumor heterogeneity, which is related to tumor proliferation, hypoxia, angiogenesis and necrosis. Increased homogeneity in colorectal cancer was related to a poor prognosis, while increased heterogeneity in oesophageal cancer and gastric cancer was associated with a poor prognosis [45–48]. Entropy and uniformity are common texture parameters, and higher entropy and lower uniformity reflected increased tumor heterogeneity [49]. However, a large number of texture parameters related to tumor aggressiveness have not been well studied.

The present study showed that AFP, PLR, largest tumor size, MVI and the radiomics signature were independent risk factors for OS. In agreement with the previous study, these clinicopathologic factors are known to be effective predictors of the clinical outcome [50–53].It has also been noted that inflammatory markers were related to HCC aggressiveness, and in our study, PLR was included in the final model [54]. However, the tumor number and tumor differentiation were not associated with survival status in our study. The possible reasons for this are the limited cases of multifocal lesions and the short follow-up time in the present study. Furthermore, in the subgroup analysis, patients in the high-risk group tended to show higher AFP, higher AST, higher PLR, larger tumor size, more hemorrhage, more intraoperative transfusion, the presence of MVI and an incomplete tumor capsule, which are demonstrated prognostic indicators of HCC [38, 39, 55, 56], indicating the potential association between the radiomics signature and clinicopathologic factors. Therefore, texture parameters are linked to clinicopathologic factors, which could assist clinicians in prognostic evaluation.

We established a combined nomogram that incorporates clinicopathologic factors and a radiomics signature for prognostic prediction at the individual level. The results indicated that the radiomics nomogram showed improved predictive accuracy over the clinicopathologic nomogram, which indicates that the radiomics signature can provide additional prognostic and biologic information; this is consistent with previous studies. Meng et al. [57] performed a study in 108 consecutive patients with locally advanced rectal cancer, and the results implied that the combined model (C-index = 0.788) exhibited improved predictive ability of the 3-year disease-free survival compared with the radiomic (C-index = 0.767) and clinicoradiologic models (C-index = 0.644). Li et al. [58] explored the prognostic value of radiomics in 181 patients with gastric cancer following curative resection and revealed that the radiomics nomogram (C-index = 0.82) showed better predictive ability than the clinical nomogram (C-index = 0.71) and the radiomics signature (C-index = 0.74). Based on the results of our study, aggressive precautions can be taken in patients with predicted poor prognoses, which would facilitate the effective therapeutic management of these patients and reduce the risk of recurrence.

The limitations of this study are as follows: (1) this study is a retrospective single-center study with a small sample size, and the results of the study are limited. In addition, the model was only verified internally and lacked external validation. (2) Contrast-enhanced CT was used in our study, whereas contrast-enhanced MRI can capture more microstructural characteristics of the tumors and may provide more comprehensive information pertaining to tumor heterogeneity [59]. (3) In our study, only the largest cross-section of a lesion in the portal phase was analyzed, while whole tumor analysis in both the arterial and portal phases may improve the efficiency of survival prediction in individuals with HCC. (4) Although the ROIs were derived from manual segmentation by two radiologists, their subjective bias could not be completely eliminated. Therefore, further studies are warranted to confirm our results.

## Conclusion

In conclusion, the radiomics signature provided a quantitative method for the assessment of survival status in patients with HCC after hepatectomy. The patients with high Rad-scores may experience a higher risk of recurrence and metastasis. Moreover, the radiomics nomogram that integrates clinicopathological factors and the radiomics signature may serve as an effective tool to guide the individualized management and tailored follow-up of HCC patients. In the future, multicenter prospective studies are needed to further investigate the potential value of the radiomics signature in clinical practice.

## Supplementary Information

**Additional file 1: Table S1.** Texture features used in the study.

## Data Availability

The datasets generated during and/or analyzed during the current study are available from the corresponding author on reasonable request.

## Abbreviations

HCC: Hepatocellular carcinoma
TA: Texture analysis
CT: Computed tomography
LASSO: Least absolute shrinkage and selection operator
Rad-score: Radiomics score
ROC: Receiver operating characteristic
OS: Overall survival
C-index: Concordance index
DCA: Decision curve analysis
AFP: α-fetoprotein
PLR: Platelet-to-lymphocyte ratio
MVI: Microvascular invasion
BCLC: Barcelona Clinic Liver Cancer
TACE: Transcatheter arterial chemoembolization
HBV-DNA: Hepatitis B virus deoxyribonucleic acid
DICOM: Digital Imaging and Communications in Medicine
ROIs: Regions of interest
ASA: American Society of Anesthesiologists
BMI: Body mass index
ALT: Alanine transaminase
AST: Aspartate aminotransferase
NLR: Neutrophil-to-lymphocyte ratio
