# Supplementary material for: A radiomics nomogram for the prediction of overall survival in patients with hepatocellular carcinoma after hepatectomy
Source: Cancer Imaging. 2020 Nov 16;20:82. doi: 10.1186/s40644-020-00360-9 (PMC7667801; doi:10.1186/s40644-020-00360-9)
Supplement: Supplementary file 1 — Additional file 1: Table S1. Texture features used in the study. [file 40644_2020_360_MOESM1_ESM.docx]

**Supplementary table S1**

**Texture features used in the study.**

| Algorithms | Description | Texture Features |
| --- | --- | --- |
| histogram features (9) | the distribution of pixel intensity without spatial information | Mean (histogram’s mean), |
|  |  | Variance (histogram’s variance), |
|  |  | Skewness (histogram’s skewness), |
|  |  | Kurtosis (histogram’s kurtosis), |
|  |  | Perc.01% (1% percentile), |
|  |  | Perc.10% (10% percentile), |
|  |  | Perc.50% (50% percentile), |
|  |  | Perc.90% (90% percentile), |
|  |  | Perc.99% (99% percentile) |
| co-occurrence matrix (220) | the frequency with which two pixel intensities co-occur within a pre-defined spatial distance around each pixel | AngScMom (angular second moment), |
|  |  | Contrast (contrast), |
|  |  | Correlat (correlation), |
|  |  | SumOfSqs (sum of squares), |
|  |  | InvDfMom (inverse difference moment), |
|  |  | SumAverg (sum average), |
|  |  | SumVarnc (sum ariance), |
|  |  | SumEntrp (sum entropy), |
|  |  | Entropy (entropy), |
|  |  | DifVarnc (difference variance), |
|  |  | DifEntrp (difference entropy) |
|  |  | features computed for 4 directions: (a, 0), (0, a), (a, a), (a, -a) and 5 distances: a = 1, 2, 3, 4, 5, between image pixels |
| run-length matrix (20) | counts of pixel runs with the specified gray-scale level and length | RLNonUni (run length nonuniformity), |
|  |  | GLevNonU (grey level nonuniformity), |
|  |  | LngREmph (long run emphasis), |
|  |  | ShrtREmp (short run emphasis), |
|  |  | Fraction (fraction of image in runs), |
|  |  | features computed for 4 various directions: vertical, horizontal, 45-degree and 135-degree directions |
| autoregressive model (5) | description of texture based on the statistical correlation between neighboring pixels | Teta1 (parametr θ1) |
|  |  | Teta2 (parametr θ2) |
|  |  | Teta3 (parametr θ3) |
|  |  | Teta4 (parametr θ4) |
|  |  | Sigma (parametr σ) |
| wavelet transform (16) | the energy of the wavelet-subband coefficients | WavEn (wavelet energy) |
|  |  | feature computed at 4 scales: s-1, s-2, s-3, s-4 within four frequency bands: the approximation subband (LL), the horizontal detail subband (LH), the vertical detail subband (HL), and the diagonal detail subband (HH). |
